# Feature Sensing and Robotic Grasping of Objects with Uncertain Information: A Review

**DOI:** 10.3390/s20133707

**Published:** 2020-07-02

**Authors:** Chao Wang, Xuehe Zhang, Xizhe Zang, Yubin Liu, Guanwen Ding, Wenxin Yin, Jie Zhao

**Affiliations:** State Key Laboratory of Robotics and Systems, Harbin Institute of Technology, Harbin 150001, China; wangchaohit@hit.edu.cn (C.W.); zhangxuehe@hit.edu.cn (X.Z.); zangxizhe@hit.edu.cn (X.Z.); 17B908042@stu.hit.edu.cn (G.D.); 18B908088@stu.hit.edu.cn (W.Y.); jzhao@hit.edu.cn (J.Z.)

**Keywords:** uncertain objects, geometric uncertainty, physical uncertainty, feature sensing, robotic grasping

## Abstract

As there come to be more applications of intelligent robots, their task object is becoming more varied. However, it is still a challenge for a robot to handle unfamiliar objects. We review the recent work on the feature sensing and robotic grasping of objects with uncertain information. In particular, we focus on how the robot perceives the features of an object, so as to reduce the uncertainty of objects, and how the robot completes object grasping through the learning-based approach when the traditional approach fails. The uncertain information is classified into geometric information and physical information. Based on the type of uncertain information, the object is further classified into three categories, which are geometric-uncertain objects, physical-uncertain objects, and unknown objects. Furthermore, the approaches to the feature sensing and robotic grasping of these objects are presented based on the varied characteristics of each type of object. Finally, we summarize the reviewed approaches for uncertain objects and provide some interesting issues to be more investigated in the future. It is found that the object’s features, such as material and compactness, are difficult to be sensed, and the object grasping approach based on learning networks plays a more important role when the unknown degree of the task object increases.

## 1. Introduction

Robots have been used in industrial manufacturing for decades and are being applied in more flexible scenarios with the improvement of robotic capability to manipulate varied objects [1,2,3]. In particular, the robotic capabilities of sensing and manipulating unfamiliar objects spawn more applications and enormous economic benefits. For instance, robots used in restaurants improve the efficiency of food delivery and also reduce labor cost [4]. Moreover, the successful applications of domestic robots help people to handle some housework to save people’s time [5], and robots in factories perform repetitive tasks quickly for a long time and reduce the loss caused by the worker’s fatigue [6].

For objects with certain information, their features—such as position, shape, pose, rigidity, etc.—are well known previously to the robot before grasping. According to these known features, a reasonable motion trajectory is planned easily and then a grasping strategy is developed, such as form-closure grasp [7] and force-closure grasp [8]. An important issue is that approaches designed for objects with certain information cannot be applied directly to objects with uncertain information. For instance, the form-closure grasping fails to handle objects with uncertain shapes for the limitation of degrees of freedom [9]. Similarly, the force-closure grasp relies on the contact forces that depend on the rigidity of the object [10]. It means that objects with a rigidity uncertainty are difficult to be grasped through the traditional force-closure approaches. Moreover, as the unknown object may have many kinds of uncertain features, the assumptions, such as simplified contact models [11] and rigid body modeling [12], are not suitable to deal with this kind of object. On the contrary, data-driven grasping—i.e., a learning-based approach—is more inclusive to the object’s unknown nature and receives more and more attention from researchers.

**Figure 1:**
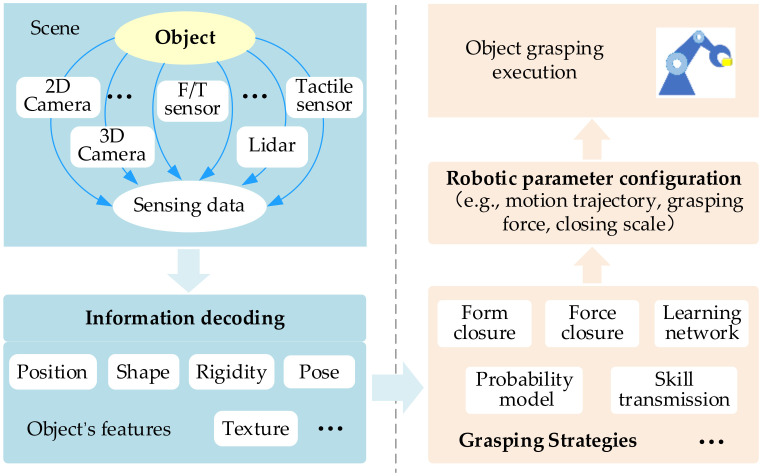
Pipeline describing feature sensing and robotic grasping.

In order to grasp the object, the robot needs to complete two processes, which are feature sensing and robotic grasping. On the left side of Figure 1, feature sensing is divided into two steps—sensor sensing and feature identification at different phases. Different types of sensor are used to detect the object to obtain the original data. Furthermore, object features—such as position, pose, shape, etc.—are estimated by decoding these sensing data. On the right side of Figure 1, the robotic grasping is realized through varied grasping strategies, which are based on various metrics, such as grasper structure, grasping force, grasper form, success probability, etc. Based on object features from feature sensing, the grasping strategy generates parameter configurations for the manipulator and grasper, and then the robot grasps the object with the given robotic configurations.

In this paper, we review the latest advances in the topic of the feature sensing and robotic grasping of objects with uncertain information. There have been many surveys covering the topics of object grasping or object manipulation, such as 3D object grasping synthesis [13], data-driven grasping synthesis [14], deep learning applications in robotic grasping detection [15], and the robotic manipulation and sensing of deformable objects [10]. Sahbani et al. [13] reviewed computational algorithms for generating 3D object grasping, particularly the analytical as well as empirical grasp synthesis approaches. Bohg et al. [14] focused on the data-driven grasp synthesis and divided the approaches into three groups for known, familiar, or unknown objects. Caldera et al. [15] summarized the current state-of-the-art approaches in regard to the application of deep learning methods to generalized robotic grasping and discussed the effect that the deep learning approach has on the overall performance of robotic grasping detection. Sanchez et al. [10] presented recent work on the robotic manipulation and sensing of deformable objects, where the reviewed deformable objects are classified into four categories, which are cloth-like objects, linear objects, planar objects, and solid objects. Following these reviews, the field of object grasping has made great progress.

**Figure 2:**
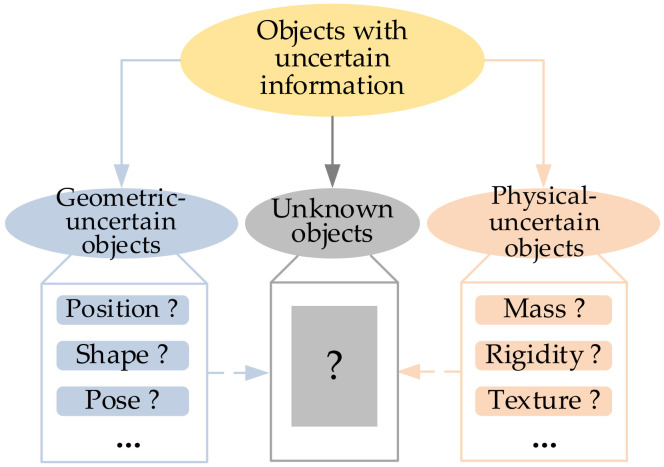
Classification of objects with uncertain information.

Our work focuses on the recent state-of-the-art approaches in the robotic community to address the sensing and grasping of objects with uncertain information. Although the classification of objects may vary with the type of uncertain information, objects with uncertain information could be classified considering their geometric features and physical features. Objects could be categorized based on the category of uncertain parameters. For instance, an object with an uncertain shape or position could be seen as a geometric-uncertain object because the shape or position could be described with geometric parameters, such as length and coordinates. Similarly, an object with an uncertain mass or rigidity could be regarded as a physical-uncertain object, as the mass or rigidity belongs to physical parameters. Based on these criteria, the reviewed approaches are classified into the following three main categories, as shown in Figure 2.

(1) Geometric-uncertain objects: This type of object usually lacks some generalized geometric information that not only includes objects’ geometric information but also the necessary geometric information for the grasp. For instance, the object’s intrinsic shape and the global position of the object both belong to the generalized geometric information. This type of object is often seen in service and industrial scenarios, such as the delivery of multi-shape fruits or picking up multitype parts within a box.

(2) Physical-uncertain objects: This type of object commonly is short of some physical information, particularly the object’s physical property, which may be used in object grasping. For instance, the mass and rigidity of the object, which could be utilized to generate a feasible grasping configuration for the robot, are significant physical properties for objects to be operated. This type of object is also commonly found in unfamiliar service scenarios or industrial scenarios with multi-unfamiliar objects, like food sorting and garbage classification.

(3) Unknown objects: Compared to geometric-uncertain objects and physical-uncertain objects, the partial geometric and physical information of this type of object may be both uncertain. This kind of object is usually referred to as objects that are not common in daily life and often coincide with an unknown environment.

This paper is organized as follows. Section 2 provides an overview of the feature sensing and robotic grasping of geometric-uncertain objects, which include position-uncertain objects, pose-uncertain objects, and shape-uncertain objects. Section 3 concerns of sensing and grasping approaches for physical-uncertain objects. Particularly, it includes sensing approaches for the object’s mass, rigidity, and texture and grasping approaches for rigid objects and deformable objects, respectively. Section 4 introduces the localization and feature identification of unknown objects and summarizes several typical approaches for unknown object grasping. Finally, Section 5 discusses the pros and cons of the reviewed approaches for each type of uncertain object and gives some interesting problems to be more investigated in the future.

## 2. Geometric-Uncertain Objects

In this section, we review sensing and grasping approaches used on geometric-uncertain objects, such as position-uncertain objects, pose-uncertain objects, and shape-uncertain objects. Although there are other geometric features for objects, they may be not very important for robotic grasping or could be replaced with the given features. Most of the research, particularly for the robotic grasping process, focus on these features of objects. For instance, the robot localizes the object based on the position information, calculates the feasible grasping points based on the shape or pose information, and then grasps the object with a planned movement and a reasonable pose of the grasper.

### 2.1. Position Detection

In service and industrial applications, particularly food handling and packaging, different types of sensors—such as 2D cameras, 3D cameras, and depth sensors—are commonly used for object localization [16]. For instance, a 2D camera obtains the object image and identifies the object through image processing algorithms [17]. The 2D image could achieve the plane localization of the object, while it is difficult to obtain the space coordinate of the object. Based on this consideration, the distance sensor is added into 3D cameras to detect the object’s depth information. Following these researches, the point cloud data [18] from depth sensors are also used for object localization.

Research into position detection based on vision has been highly reliable after decades of development. Particularly, camera calibration technology [19,20,21] enables a robot to obtain an object’s position with higher accuracy. The RGB pixel values of the object are easily obtained by RGB cameras, and the spatial coordinates of the object could be calculated by a Decision Tree and Decision Fusion [22]. With the development of machine learning techniques, neural networks are gradually used in the field of object recognition. The trained Convolutional Neural Network could be used to identify objects in multi-cluttered 2D images and test the performance of object recognition. In the paper [23], a general CNN-based multi-modal learning framework is proposed for object recognition. This framework constructs the depth CNN layer for color data and depth data and connects them by a specially designed multimodal layer. This approach could not only segment the object from clutter but also estimate the object’s position. Additionally, the color data and depth data from the RGB-D camera could be processed by Hierarchical Matching Pursuit (HMP) [24] to localize the object. The HMP is a multi-layer sparse coding network, and the data of the RGB-D camera are presented as abstract features to facilitate the SVM to obtain a better recognition result. The discriminative features extracted from RGB-D images are used to encode the RGB-D point cloud data, and the object could be recognized by Hierarchical Cascaded Forests [25] and Recurrent Convolutional Fusion [26].

Moreover, the spatial-temporal features of objects could be extracted from the point cloud data, which are significant information for object recognition and classification [27,28,29,30]. This process consists of three main periods: (a) depth sensor data filtering, (b) object segmentation and noise reduction, and (c) object recognition and classification using CNN. The point cloud data can not only be used alone but can also combine with RGB images for object localization. A sensory-fusion framework, Multi-View 3D Networks, is proposed [31]. This framework takes both point cloud data and RGB images as inputs to detect 3D objects.

### 2.2. Shape Identification

The object’s shape is an important reference for robotic grasping. The object’s shape could be used to calculate the feasible grasping points. For uniform objects, the object’s shape could be previously provided for the robot—i.e., the shape could be seen as the known feature. However, as for nonuniform objects or objects with occlusion, it would be a challenge regarding shape identification. There are two types of approaches to deal with this issue.

**Figure 3:**
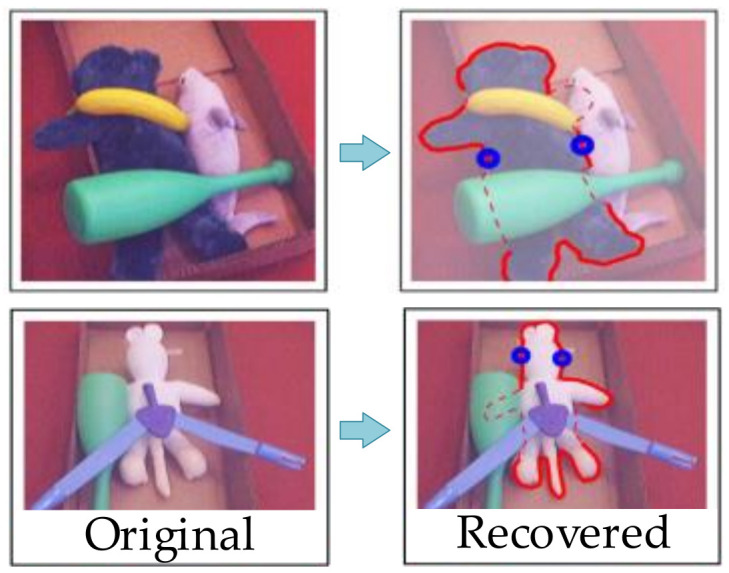
Shape identification with completed 2D boundary [33].

The first is to extract the 2D image feature of the object and then estimate the 2D shape to provide supports for robotic grasping. Generally, there are multiple objects in a single image. The first step is to partially recognize the object based on the context information and exploit edge information to estimate the object’s shape at the same time. This process has been realized by the learning-based approach [32]. Besides this, the generative model is also available. In research [33], a generative model for the object geometry was studied and could be extended to recognize the object’s shape in a cluttered image, with which the robot calculates the grasping point; the example is as shown in Figure 3.

The rest is that the object’s 3D data is used to calculate the 3D shape of the object. The advantage of this approach is that it provides rich information for the calculation of grasping points to find the best grasping point in space, particularly for unstructured objects. Chiu et al. [34] built a 3D class model to estimate the object’s 3D shape, including occluded parts, from a single image. This model is learned from a few labeled images for each class. Moreover, the 3D shape identification could be realized by learning networks. Kalogerakis et al. [35] introduced a deep architecture for the 3D shape identification of objects. This architecture combines image-based Fully Convolutional Networks (FCNs) and surface-based Conditional Random Fields (CRFs) to yield a coherent segmentation of 3D shapes. The image-based FCNs are used for efficient view-based reasoning about 3D object parts. Through a special projection layer, FCN outputs are effectively aggregated across multiple views and scales, then are projected onto the 3D object surfaces. At last, a surface-based CRF combines the projected outputs with geometric consistency cues to yield the 3D shape. Although their results show that this architecture is effective, there are particular cases where the training data is not enough, showing that the need for a better design of architectures and networks. Additionally, the CAD matching approach is a good choice to reconstruct and identify the 3D shape of the object. Kong et al. [36] use the dictionary of dense CAD models to reconstruct an object’s 3D shape from a single image. Firstly, the orthogonal matching pursuit is employed to rapidly choose the “closest” single CAD model in the dictionary to the projected image. Then, a novel graph embedding based on local dense correspondence is used to refine the camera position and create a dense 3D model of the object by fitting both landmarks and the silhouette. Kurenkov et al. [37] propose the DeformNet framework, which generates a 3D shape reconstruction from a single image. This network takes an image input, finds the nearest shape template from a 3D CAD database, and deforms the template to match the query image. Moreover, a new differentiable layer is introduced for 3D data deformation, and this is used in DeformNet to learn a model for 3D shape reconstruction.

### 2.3. Pose Estimation

The pose estimation of objects in the field of robotics has been a hot topic for a long time. We summarize three types of approaches for pose estimation which are based on template, voting, and learning. 

**Figure 4:**
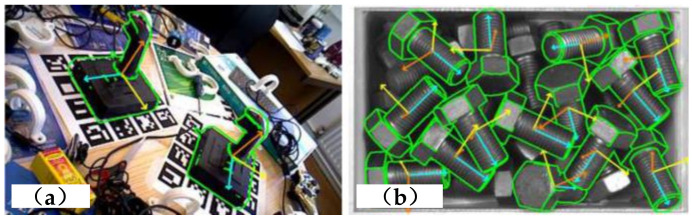
Scenarios of objects’ 6D pose estimation [40]: (**a**) tabletop scenario, (**b**) bin-picking scenario.

The first one is template-based approaches. Based on the priori template, a template-based approach, like LINEMOD [38], extracts the gradient information for matching and then estimates the object’s pose. In the paper [39], a hierarchical fragment matching approach for 3D pose estimation is presented. The Clustered Centerpoint Feature Histogram, as a descriptor, is used to compute the features of object fragments. Then, the Extreme Learning Machine classifier is applied to identify the matched segments and generate the estimated pose. In the paper [40], a template matching-based approach is proposed for the 6D pose estimation. This approach consists of three main components, PCOF-MOD (Multimodal PCOF), balanced pose tree (BPT), and optimum memory rearrangement for a coarse-to-fine search. The PCOF-MOD template is based on gradient orientation extracted from RGB images and describes the shape of object contours. These templates are integrated into BPT to reduce the search space for the 2D position and 3D pose simultaneously and make the pose estimation faster. The optimum memory rearrangement rearranges the input features so that the different types of neighboring pixels are linearly aligned. The estimated objects’ 6D poses by this approach on tabletop (Figure 4a) and bin-picking (Figure 4b) scenarios are as shown in Figure 4.

The second one is the voting-based approach. This type of approach usually uses every local feature to predict a pose and then finds the optimal result by voting. The classical approaches include the Hough Forest approach [41,42,43] and the Point Pair Features approach [44,45]. Tejani et al. [42,43] proposed a novel framework, Latent-Class Hough Forests, for 3D pose estimation in heavily cluttered and occluded scenarios. The template matching feature, LINEMOD, is adapted into a scale-invariant patch descriptor which is integrated into a regression forest using a novel template-based split function. Every tree in the regression forest maps an image patch to a leaf that stores 6D pose votes. Drost et al. [44] realized the recognition of freeform 3D objects in point clouds. Global model description is created based on oriented point pair features, which consist of all model point pair features and represent a mapping from the point pair feature space to the model. Pose recognition is performed locally using an efficient voting scheme on a reduced two-dimensional search space. Following this research, Vidal et al. [45] proposed a variation of the PPF method and won the SIXD challenge.

**Figure 5:**
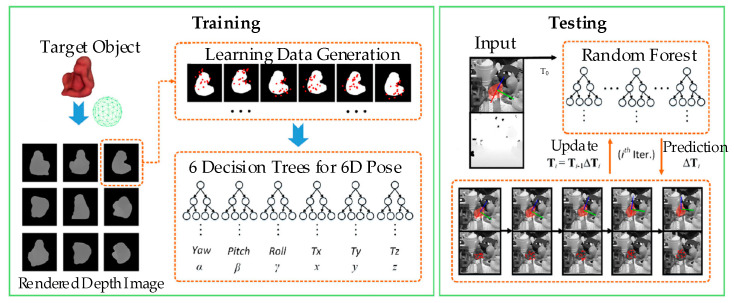
The whole pipeline of the random forest approach [46].

The last one is the learning-based approach. This approach needs a previous database to train estimation models or networks and could deal with objects in depth images and objects in color images as well. Based on the depth image, the random forest approach [46] could achieve the 6D pose estimation. As shown in Figure 5, this approach consists of two parts: training and testing. In the training process, depth images are taken as the inputs to train six trees and give the initial pose. In testing, based on the initial pose and the depth image, the forest prediction is used to refine the object’s pose. As for objects in RGB images, Georgakis et al. [47] proposed an approach for 3D pose estimation. This approach does not need to mark the object’s pose in the training phase. Through the deep quadruplet CNN, the relationship between RGB images and the rendering depth images of the CAD model is established, and then the object’s pose is estimated via the RANSAC algorithm and PnP algorithm.

### 2.4. Robotic Grasping

For geometric-uncertain objects, most physical properties could be seen as known and not considered for robotic grasping in this section. The robotic parameters are more related to the geometric features of the object, such as position, shape, and pose. The commonly used grasping approach could be described as a direct configuration-based grasping approach that grasping control is related to in the structure of the grasper.

**Figure 6:**
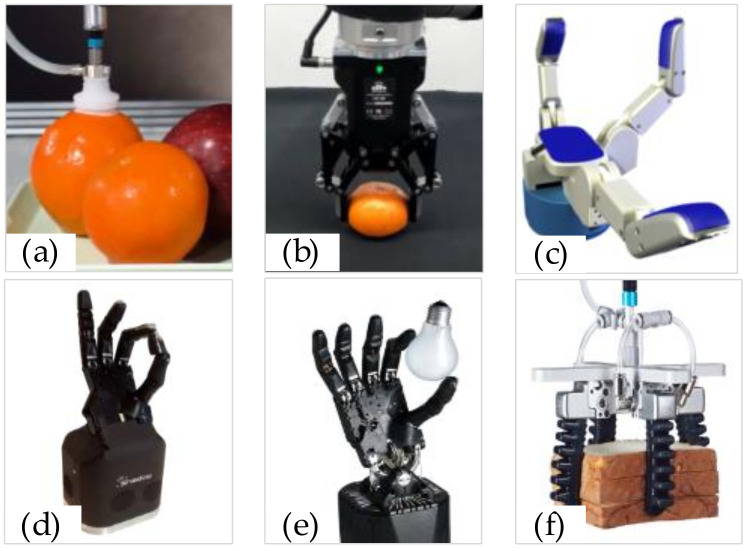
Different structures of grasper: (**a**) sucker [48], (**b**) two-fingered grasper [49], (**c**) three-fingered grasper [50], (**d**) four-fingered grasper [51], (**e**) five-fingered grasper [51], (**f**) soft grasper [48].

According to different grasping requirements, varied structures of graspers are designed, such as the sucker, the multi-fingered dexterous grasper, and the soft grasper, as shown in Figure 6. Due to having more degrees of freedom and a better shape adaptability, the multi-fingered dexterous grasper and the soft grasper are more powerful for different types of objects. The configuration of the multi-fingered dexterous grasper and the soft grasper are also more challenging compared to other graspers.

For the multi-fingered grasper, a framework including Multi-Dimensional Iterative Surface Fitting (MDISF) and Grasp Trajectory Optimization (GTO) for grasp planning is proposed [52]. The MDISF is used to search for the optimal contact region based on the position of the object. The hand configuration is optimized by minimizing the collision and surface fitting error. The optimal trajectory to reach the highly ranked grasping configuration is generated by GTO. The grasping configuration could also be generated according to the curvature of objects. In the paper [53], Calli et al. propose an active visual grasping algorithm for the hand-eye system. This algorithm models the object with the Elliptic Fourier Descriptors and uses a visual servo rule to realize the grasping configuration with curvature measurements.

**Figure 7:**
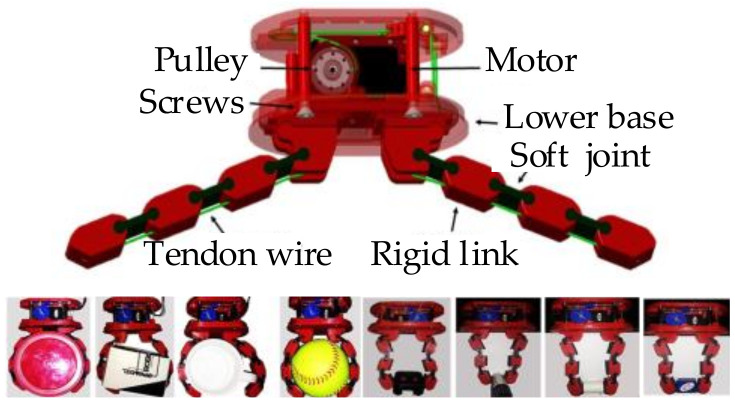
Object grasping with a soft grasper [54].

Furthermore, to overcome the problem that the soft grasper is difficult to control accurately, an efficient mathematical representation of soft fingers based on screw theory is proposed [54]. As shown in Figure 7, this grasper is underactuated; tendon-driven; and consists of two flexible fingers, whose configuration could be changed according to the object’s shape. The grasping state is detected through rolling soft fingertips, and the grasping force and the grasper pose are changed by adjusting the control parameters [55]. However, there also exist some constraints—such as contact reachability, object restraint, force control, etc.—for a specific manipulating task. As a result, Rosales et al. [56] present a kinematic formulation for the grasping synthesis problem. This formulation could generate an optimal grasping configuration by considering these constraints.

## 3. Physical-Uncertain Objects

For the process of object grasping, the robot is required to localize the object and find the feasible grasping points at first. Next, the robot needs to grasp and manipulate the object regarding the desired task. In industrial scenarios where heavy and light objects are usually manipulated alternately, it is not reasonable to grasp the object only with geometric features. For instance, variable mass parts usually require different grasping forces, otherwise it is easier for the grasp to be unsuccessful or destructive. Similarly, in some service scenarios where the robot needs to deal with a set of deformable objects, this is an unlikely task without the rigidity information of the object. 

Furthermore, for a scenario with physical-uncertain objects, the grasping approach for geometric-uncertain objects does not work anymore. Generally, the physical properties of the object are identified firstly, and then the robotic grasping parameters are configured based on these properties. In the process of object grasping, the physical parameters that are commonly concerned include mass, rigidity, and texture. In this section, we review approaches used on physical-uncertain objects, such as mass-uncertain objects, rigidity-uncertain objects, and texture-uncertain objects. There has been a lot of research on these properties, and here we have collected and sorted it out. 

### 3.1. Mass Estimation

The accurate estimation of an object’s mass is a big challenge for robots, as robots are unlikely to use the commonly used method of static weighing to obtain the object’s mass. Usually, force/torque sensors or tactile sensors are used to detect an object’s mass. For instance, Kubus et al. [57] used measurements of the force and torque from a sensor on the wrist as well as measurements of angular velocity, linear, and angular accelerations to estimate an object’s mass. Further, Petković et al. [58] designed an adaptive underactuated compliant grasper with distributed compliance and built a prediction model for the mass estimation of the object in relation to sensor stress. In addition to the one-dimensional grasping force, the mass of the in-hand object could also be calculated based on the 3D force vector [59] from tactile sensors on fingertips. Due to the limitation of the generalization ability of the estimation model, the detection accuracy is often reduced when the new object’s mass is detected.

**Figure 8:**
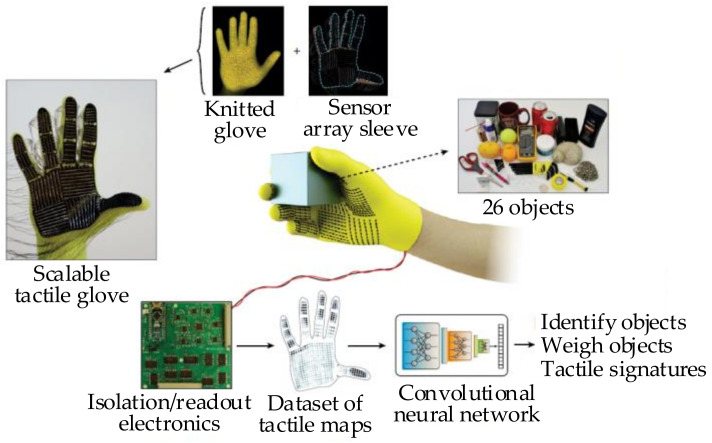
The Scalable Tactile Glove (STAG) as a platform to learn from the human grasp [60].

Moreover, deep learning also has applications in the mass detection of objects. In the paper [60], a Scalable Tactile Glove (STAG) covered with 548 tactile sensors is designed, as shown in Figure 8. This STAG acquires the normal force recorded by each sensor and builds a tactile dataset. The mass is estimated through a ResNet-18 architecture [61] based on the data from tactile sensors. This type of approach has a strong dependence on datasets, and it usually needs to spend a lot of time to train the learning network.

In addition, the visual approach is also used to detect the object’s mass. The principle of the visual approach is establishing the relationships between the object’s visual information (object geometry, RGB images, depth images, point clouds, etc.) and the object’s mass properties, and then calculating the object’s mass based on the real-time data from visual sensors. The regression model, like the geometric outline-mass model [62], the volume-mass model [63,64], etc., is a commonly used way to identify an objects’ mass. The mass detection precision of this type of approach is not very high and mainly depends on the accuracy of the mapping model and the reliability of the visual detection.

### 3.2. Rigidity Prediction

Rigidity is one of the most important properties of objects. An object with an uncertain rigidity is difficult to manipulate successfully, as the object may be distorted by the grasping force. Most of the research is directly aimed at rigid objects or deformable objects to give the corresponding grasp and manipulation strategy. There is little research on objects’ rigidity detection for object grasping, which severely restricts the autonomous task ability of the robot to grasp objects.

For a robot, it is hard to accurately obtain the rigidity parameter of the object before the robotic grasping. The application of sensors in robotic manipulation still has many limitations. Comparatively, it is feasible that the rigidity degree of an object is predicted to distinguish rigid objects and deformable objects, and then the manipulation strategy is given according to the classification result. In the paper [65], Zang et al. proposed a motion analysis-based approach for detecting an object’s rigidity. This approach distinguishes rigid objects and nonrigid objects by coupling motion estimation and optic flow matching. Moreover, tactile sensors are also used to classify objects. Drimus et al. [66,67] designed a flexible tactile sensor for the classification of rigid objects and deformable objects. The flexible tactile-sensor array is used to acquire the rigidity information of the object. The array of tactile information is denoted as a time series of features and used as the input for the k-nearest neighbor classifier to classify the various rigid objects and deformable objects. After the classification of the object is given, the manipulations could be carried out according to varied grasping approaches for rigid objects and deformable objects.

### 3.3. Texture Detection

Texture detection is an important part of object recognition and also plays an important role in robotic grasping. As early as 1992, higher order statistics were applied to detect and classify random textures [68]. Satpathy et al. [69] introduced two classes of edge-texture features, which are the Discriminative Robust Local Binary Pattern and Ternary Pattern. These edge-texture features make better recognition effects on objects, particularly in light and dark backgrounds. Afterward, an online texture rendering model that deals with low-texture and high-light objects is established [70]. The dynamic template in continuous 6DoFs pose space is created based on the texture rendering model. This dynamic template could be used to handle the 3D object tracking problem and the partially occluded problem.

For a cluttered environment, the specific texture plays a significant role in separating the object from the background. In the paper [71], a texture segmentation approach was proposed based on the parametric active contour model. It calculates the gray-level co-occurrence matrix and co-occurrence energy of the region inside and outside of the dynamic contour. The texture contour is determined while the co-occurrence energy is in the maximum state. Moreover, texture recognition could also be realized by the fusion of tactile and visual images. Luo et al. [72] proposed a novel fusion approach, Deep Maximum Covariance Analysis (DMCA), to learn a joint latent space for sharing features via vision and tactile sensing. The features of visual images and tactile data are learned by deep neural networks. The maximum covariance analysis is used to pair the learned features. In the paper [73], the progress of the texture representation in the past two decades is summarized, and the challenges for future research are also discussed.

### 3.4. Robotic Grasping

Applications for the robotic grasping of rigid objects and deformable objects frequently exist in industrial and service scenarios. Particularly, more applications of deformable objects in industrial and service scenarios can be found in [74,75]. An object’s features, such as mass, rigidity, and texture, can help the robot to set a feasible grasping force to grasp the object. This kind of approach is mainly used when there is no special classification for the object. Normally, the object could be divided into rigid objects and deformable objects according to the stiffness characteristics. For rigid objects, the human could teach robots how to grasp objects and manipulate them or plan the robotic grasping based on tasks. As for deformable objects, robotic grasping is more difficult to implement because of undesired deformation during manipulation. The following are grasping approaches for rigid objects and deformable objects.

#### 3.4.1. Robotic Grasping of Rigid Objects

**Figure 9:**
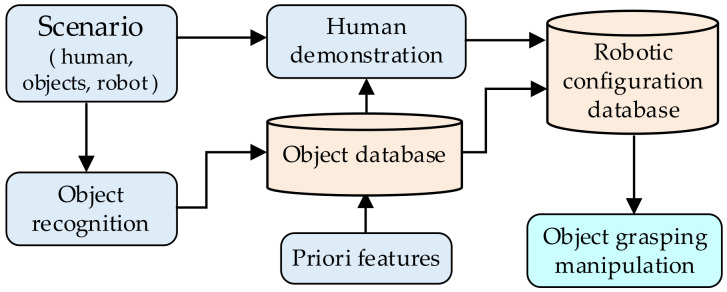
Functional flowchart of object grasping by learning from demonstration (LfD).

As for rigid objects, learning from demonstration (LfD) is a popular approach for a robot to improve its capability of object manipulation. This approach enables the robot to acquire new skills through leveraging demonstrations offered by human operators [13,76]. As shown in Figure 9, the robotic grasping configuration learned from human demonstrations and the object’s features are also taken into account. The object’s sensing information and the priori features are combined to obtain the object’s database which provides basic data for robotic configuration. Further, the robot observes the grasp and manipulation of rigid objects by the human, and the kinematic parameters of the moving process are stored. Finally, the robotic configuration database is built and guides the robot to finish the robotic grasping of the object.

The grasping strategy—such as grasping type, thumb placement, and direction, etc.—is extracted from human demonstration and then integrated into the grasping planning procedure. It is crucial to generate a feasible grasp concerning object features and manipulation tasks [77]. However, there will be some mistakes, as the human operator gives an imperfect demonstration because of the unintentional operator error in the kinesthetic teaching demonstration. To solve this problem, Mueller et al. [76] improved the LfD and proposed a novel algorithm, which is Concept Constrained Learning from Demonstration (CC-LfD). The CC-LfD not only realizes the robust skill learning but also finishes the skilled repair that incorporates the annotations of conceptually grounded constraints during live demonstrations of the LfD. Furthermore, Welschehold et al. [78] proposed an RGB-D observation-based demonstration approach. This approach prevents the requirement of accurate knowledge about the interactions between the robot and the object, which also eliminates the unintentional operator error. In order to increase the robustness of LfD, Van et al. [79] designed a GraspNet for object detection from merely a single demonstration. The GraspNet is based on a convolutional neural network and can be rapidly fine-tuned for a new demonstration. Based on data from previous demonstrations, the training time is decreased further.

In addition, the skill-based programming approach is an effective way to reduce the requirements of operators and the difficulty of programming. This approach is divided into three layers, which are the primitive layer, skill layer, and task layer [80]. The primitive level is to describe the capability of the robotic system into simple and intuitive symbolic units, such as the movement of the manipulator and the operation of the gripper. The skill layer consists of numerous skills that transform the world from an initial state to a goal state based on the specified prior parameters, such as the perception skill of the external environment for decision and the pick and place skill with provided objects and place poses. The task layer is the topmost layer of the operational abstraction and combines the required skills to achieve the objective. With skill-based programming, the robot is able to finish varied object manipulation, such as grasping, picking, assembly, and so on [81,82]. Huang et al. [83] presented a robotic system with a library of assembly skills. These skills are acquired by machine learning and can be reused for different furniture sets. Herrero et al. [84] combined skill-based programming with a state machine and improved the skills of interaction and communication with humans. The skill-based programming approach eases the robot program generation and is helpful for the robot to grasp the object and complete the task, particularly in a human–robot collaborative workspace.

**Figure 10:**
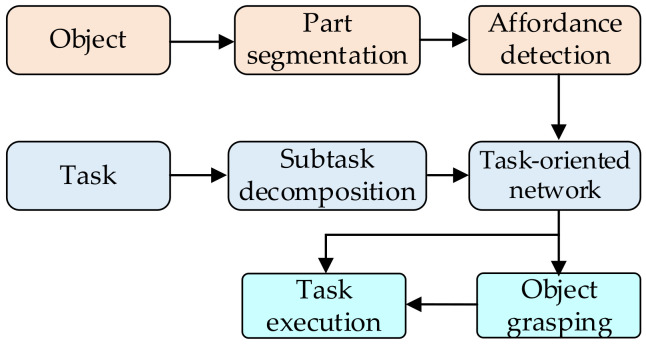
Functional flowchart of object grasping based on the task.

Moreover, the task oriented-based grasping approach is another popular option. The robot is not only required to grasp the object steadily but also to complete a specific task, such as handling, placing, parts assembly, etc. To achieve this, two processes are followed in parallel, as shown in Figure 10. For one thing, the functional meaning of the object part is reasoned, and the object is separated into several parts based on their functional meaning. The features of each object part are detected and used to generate the particular affordance for each part. For another, according to the robotic requirements of the special task, the task could be decomposed into a serial of continuous subtasks. It could be seen as a manipulation policy for the task-oriented network, and then the task-oriented network is established based on the learning network. Finally, the task-oriented network guilds the robot to finish object grasping and task manipulation.

**Figure 11:**
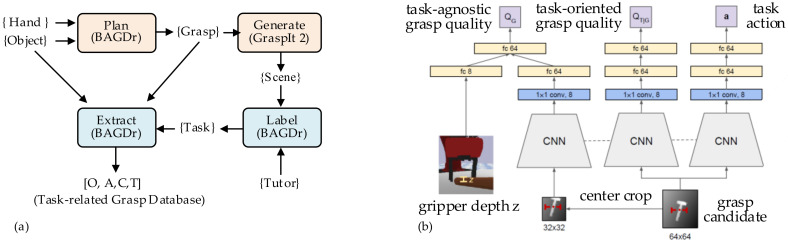
Examples of task oriented-based grasping approaches: (**a**) the schematic diagram for generating a task-related grasp database [86], (**b**) Task-Oriented Grasping Network [88].

Lakani et al. [85] proposed an RGB-D part-based approach for task performance. The affordances are detected and associated with parts of the object. As these affordances are related to the task, the task could be executed directly on the object. Furthermore, several kinds of robotic tasks are tested, such as grasping, pouring, scooping, cutting, striking, and placing. In addition, an object grasping system—Box Approximation, Decomposition, and Grasping (BADGr) [86]—is used to generate a stable grasp for a hand-object pair. As shown in Figure 11a, the stable grasps are labeled and saved into the task-related grasping database for training and testing. The affordance function is defined by basic grasping metrics and extends the grasping quality metric to task-oriented grasps [87]. This task-based grasping matrix can not only guild the grasping process, but also evaluate the grasping quality of task completion.

To establish a closer relationship between object grasping and task manipulation, Fang et al. [88] built a Task-Oriented Grasping Network (TOG-Net) to jointly optimize the task-oriented grasp and the manipulation policy. As shown in Figure 11b, the inputs of TOG-Net are two crops of depth images and the sampled grasper depth. The task-agnostic grasp quality, conditioned task-oriented grasping quality, and manipulation actions are predicted through TOG-Net. As the typical movement is associated with a particular task, the previous sensor experiences could be used in the predictive model for subsequent task execution [89]. The grasping capability could be enhanced through leveraging the semantic object parts [90]. The pre-grasp configurations are reasoned with respect to the intended task. The object-task affordances and object-task ontologies are employed to encode rules for generalizing over similar object parts and object-task categories. 

#### 3.4.2. Robotic Grasping of Deformable Objects

Deformable objects also have many applications in industrial, domestic, and service scenarios. The categories of deformable objects could be given based on their geometry. For instance, Sanchez et al. [10] classified deformable objects in domestic and industrial applications into four main categories, which are cloth-like objects, linear objects, planar objects, and volumetric objects. Moreover, Saadat et al. [74] introduced the automatic manipulation of three categories of deformable objects in industrial applications, including linear objects, sheet objects, and three-dimensional objects. As noted previously, deformable objects could roughly be categorized into three categories including linear objects, planar objects, and 3D objects.

**Linear objects:** This type of object is often smart enough to withstand one-dimensional forces, such as cables, springs, beams, and ropes. The robotic grasping of these objects is a major industrial problem. In the case of robotic grasping with ropes, the two main desired robotic tasks are knotting and inserting. The robot not only needs to control the object’s deformation but also needs to complete the given tasks. Yamakawa et al. [91] studied the relationship between a knotting process and the individual skills of a robot hand, and identified loop production, rope permutation, and rope pulling skills. In the following research [92], they derived a model of the flexible rope and proposed a motion planning method. They were able to change a rope into different shapes, such as a rectangular corner and a semi-circle. As for the insertion task with ropes, Nakagaki et al. [93] observed the shape of the wire by the stereo vision and applied a force acting on the flexible wire to control the wire’s shape to ensure that the deformed wire is transformed to the straight one. Furthermore, Wang et al. [94] realized that the robot inserts a rope through a series of holes with high robustness. However, these approaches have, so far, not been applied in real situations as their test environments are simplified. The real-time performance of these approaches also needs to be improved.

**Planar objects:** The feature of this type of object is that the geometry in one dimension is considerably smaller than the other two. For instance, the thickness of the paper is negligible compared with its width and height. Cards, foam sheets, and metal sheets are considered in this category. Most of the research focuses on two types of object manipulation, including folding and picking up. In a series of studies, such as Balkcom and Mason et al. [95], Elbrechter et al. [96], and Namiki et al. [97], the robot is able to fold a paper using predefined folds, fiducial marker tracking, or the mass–spring–damper model. Although these approaches could be extended to planar object folding, more flexible manipulations are not easily performed. As for planar object grasping, the grasper needs to maintain real-time contact with the object and adapt to the object’s deformation. Gopalakrishnan and Goldberg et al. [98,99] defined the concept of deform closure, where the form closure of rigid object grasping is extended to deformable planar objects. Furthermore, the deformable parts are modeled as linearly elastic polygons with a triangular finite element mesh and given stiffness matrix. This research is based on the assumption that the elasticity is linear and the gravity is negligible. The effect of these aspects needs to be studied in the future. More analysis of grasping deformable planar objects could refer to the review [100].

**3D objects:** In its geometry, this type of object does not have extreme disparity in each dimension. Objects such as plush toys, dough-like foods, bulk material, etc., belong to this category. This type of object is more likely to produce unexpected deformation and usually requires more comprehensive information to assist with robotic grasping. Jørgensen et al. [101] captured a point cloud of deformable objects using a structured light scanner and used the pick and place strategy after analyzing the sensing data. This approach could manipulate different pork cuts by suction cups rather than fixed grippers in real-world trials. In addition, Delgado et al. [102,103] used tactile sensors to achieve the estimation of deformability degree during the grasping process and the adaptation of deformable objects with different elastic properties. They not only consider the deformation of the object but also take the object’s softness into account. Later on, they proposed an adaptable control scheme with a tactile servo to handle the manipulation tasks of deformable objects. In addition, the physically based model is also significant for deformable object grasping. For instance, the Kelvin–Voigt model [104] that describes the contact relationship between the grasper and the object could enable the grasper to lift the deformable object with a minimum force. The finite-element model [105] could approximate the deformation of the object caused by the grasper. Although these models work well in the simulation environment, their usability in real life still needs to be further explored, because the real model is usually more complicated and the accurate model is difficult to obtain.

## 4. Unknown Objects

In this section, we review the approaches used on unknown objects of which some geometric and physical features both are uncertain. This type of object often exists in unknown environments or multiple object scenarios with occlusion. The first work for the robot is to search and localize the object, because the object’s information is hardly determined, particularly in environments with multiple uncertain objects or occlusion. The next work is for features—such as pose, shape, mass, rigidity, etc.—to be identified for the robotic grasping. The approach used for geometric-uncertain objects and physical-uncertain objects could also be applied to identify the geometric and physical information of unknown objects.

Moreover, the robotic grasping is usually complicated, as the parameters of the object have too much uncertainty. These uncertainties severely limit the robot’s ability to manipulate the object. From the perspective of applications and task requirements, the geometric and physical parameter identification of unknown objects could be collectively referred to as feature identification without explicit classification. Due to the uncertainty impact, typical grasping approaches—such as form-closure approaches and force-closure approaches—are difficult to apply. The approach based on skill learning and transferring by learning networks is more popular. In some way, the grasping experience of familiar objects improves the grasping robustness of unknown objects.

### 4.1. Search and Localization

As for unknown objects, one of the most challenging problems for object grasping is how to obtain an accurate position with regard to the robot. Generally, sensors—such as 2D cameras, 3D cameras, depth sensors, and tactile sensors—are used for the search and localization of unknown objects. Notwithstanding, the 2D camera does not acquire all the feature information of unknown objects, especially objects with occlusion, and it works for the rough localization of the object. In contrast, the 3D camera not only obtains the plane feature information of the object but also acquires the spatial position information. As a result, the unknown object recognition by 3D cameras has become a research hotspot in recent years [106]. Compared with passive sensors, such as cameras, lidars, etc., the tactile sensor inspired by human touch acquires object feature information by active contact perception. The information obtained by tactile sensors is the closest to the real features of the object. 

**Figure 12:**
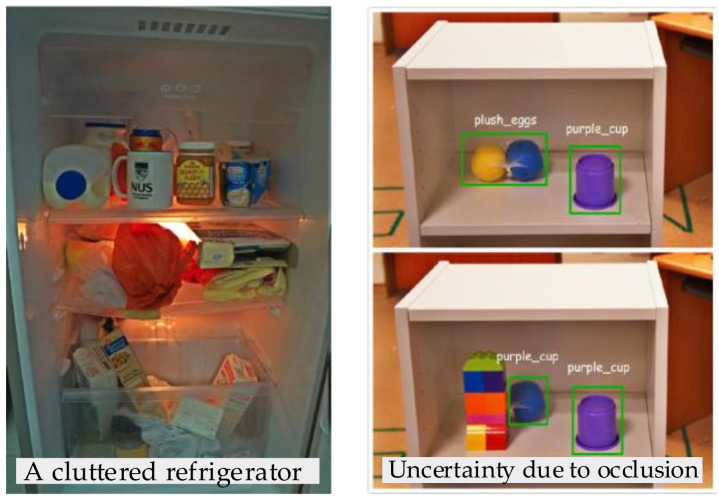
Object searching in a cluttered environment [108].

According to the characteristics of varied sensors, approaches for the search and localization of unknown objects are classified into three categories, which are image-based approaches, point cloud-based approaches, and tactile perception-based approaches. The first approach is that the object is detected and segmented from the 2D image, and the part registration of the object is generated with the viewpoint identification. Based on Extremum Seeking Control [107], the active vision strategy is able to conduct continuous optimization backed up with mechanisms to escape from local maxima. For the clutter environment, as shown in Figure 12, the Partially Observable Markov Decision Process (POMDP) [108] is used to solve the problem of object searching. In the clutter environment, the robot needs to move objects around to acquire the information of the object, which reduces the perception uncertainty. The POMDP provides a principled planning and decision-making framework for the robot to search and move the object in a partially observable domain. In addition, the 3D point cloud data could be fused with images to localize the 3D object. Xu et al. [109] proposed a dense PointFusion architecture, where the image data and original point cloud data are independently processed by CNN and PointNet architecture. The 3D point cloud data are used as spatial anchors to predict the hypothesis and reliability of multiple 3D boxes. As the boundary box of the object is determined, the object’s position could be calculated. Notwithstanding that the image and point cloud data can estimate the position of objects, there are still residual uncertainties, especially when the important features of objects are obscured.

The third approach is that the tactile perception combined with proprioception provides the highly reliable localization information of objects. Hsiao et al. [110] presented a decision-theoretic approach to solve the localization problem. The robotic actions are guided by tactile feedback to search the object and localizes the object by selection among the parameterized set of trajectories. Vezzani et al. [111] introduced an effective Bayesian-based algorithm named Memory Unscented Particle Filter. This algorithm recursively solves the 6DoFs localizing problem in a timely way by the measurement of contact points. In addition, an active exploration approach solely based on tactile information for the robot to explore unknown workspaces is introduced in [112]. This active approach enables the robot to explore the whole workspace and cluster all the data points to acquire the points that belong to the same object into one category. Based on the category, a 3D minimum bounding box is calculated to represent the object. The geometric features of the object, such as length, width, and height, are roughly estimated by calculating the Euclidean distance, and then the coordinate of the object is calculated. Although this type of approach could obtain more realistic information, the exploration efficiency is low, and the exploration accuracy is greatly affected by the sensor resolution.

### 4.2. Feature Identification

After obtaining the object’s position, the robot needs to decide how to grasp the object. Although many feature parameters of unknown objects are useful for the robotic grasping, the pose and shape of the object are more important for the grasp. These parameters determined the initial motion configuration of the robot to grasp the object. In addition, other properties concerned—such as color, mass, material, and compactness—are also discussed in this section.

#### 4.2.1. Pose Estimation

**Figure 13:**
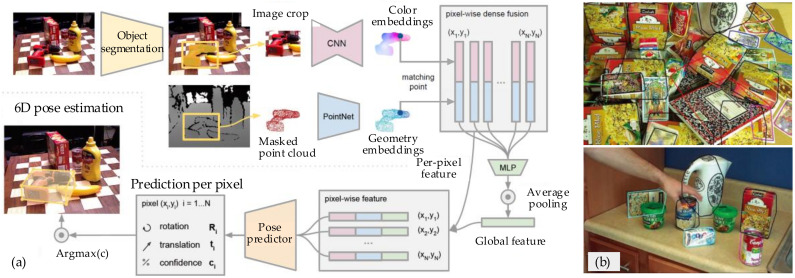
Approaches for pose estimation: (**a**) the DenseFusion architecture [113], (**b**) recognition based on the multi-object pose estimation and detection (MOPED) framework [115].

The pose estimation of unknown objects is more challenging than other objects because most information on the object is uncertain. To solve this problem, Wang et al. [113] proposed a DenseFusion architecture for 6D pose estimation based on RGB-D images, as shown in Figure 13a. This architecture processes the RGB data and depth data and then extracts the pixel-wise features for pose estimation. In addition, Tekin et al. [114] presented a single-shot approach for the pose prediction of objects in RGB images. A CNN architecture predicts the 6D pose by the PnP algorithm and realizes the real-time processing without requiring multiple periods or examining multiple hypotheses. For the problem of multi-object pose estimation, Collet et al. [115] proposed a detection framework for multi-object pose estimation and detection (MOPED). This framework addresses two main challenges, which are robust performance in complex scenarios and low latency for real-time operation. The Iterative Clustering Estimation algorithm is used to estimate groups of features that belong to the same object. Through clustering, the object hypotheses are searched within each of the groups. Then, the poses are estimated using Levenberg–Marquardt by iterations to obtain the final poses from each cluster. The examples of pose recognition are shown in Figure 13b.

#### 4.2.2. Shape Detection

In addition to the approaches mentioned in the shape detection of geometric objects, the approach based on tactile perception is also a trend at present. In the research [116], the problem of shape reconstruction from sparse tactile data is studied. The Information Gain Estimation Function combines different goals as a criterion to quantify the cost-aware information gain during exploration. This approach reconstructs the object’s shape by adding multi-oriented contacts based on criteria such as local information maximization and exploration cost minimization. Based on tactile sensors, a fast estimation criterion [117] is chosen for active contact selection, which not only considers the uncertainty of shape estimation but also takes the travel cost of contact into account. In addition, the Monte Carlo tree searching approach [118] is used to optimize the gesture sequence of the wrist and select the gesture to obtain the maximum recognition possibility. The Gaussian Process Implicit Surface Model is used to learn the shape of objects from tactile information and evaluate the estimation uncertainty. These approaches extend the research field of unknown objects’ shapes, but the timeliness and accuracy of the detection results have not been able to meet the standards required for practical applications.

#### 4.2.3. Other Properties Identification

In addition to features, such as position, pose, shape, and texture, which are commonly used in object recognition and robotic grasping, other features, such as color, material, compactness, mass, etc., need also be further considered in the process of the recognition and grasp of unknown objects. The object color is compact and computationally efficient, and particularly is effective for the recognition of occluded objects [119]. Sande et al. [120] studied the invariance properties and distinctiveness of color descriptors. The analytical invariance properties of color descriptors are explored by the taxonomy with respect to photometric transformations. In addition, Škoviera et al. [121] built a bio-inspired intelligent network named Hi-Erarchical Temporal Memory. This network recognizes the object in cluttered color images based on the information of color, texture, and shape. In general, the mass of an object could be calculated by Hooke’s law. However, the motion process of a manipulator is a multi-dimensional motion process, and it is difficult to maintain the in-hand object to carry out the one-dimensional motion state during the object manipulation. Therefore, the mass information of the object is usually acquired by an indirective estimated approach instead of calculating with force data directly. The mass estimation approach used for physical-uncertain objects could be extended to unknown objects. In the field of robotic grasping, however, not all properties are easily obtained because of the limitations of sensor technology. At present, there is no very effective approach to detect the properties of material and compactness. Therefore, more studies on sensor technologies and detection approaches to identify objects’ material and compactness need to be performed in the future.

### 4.3. Robotic Grasping

**Figure 14:**
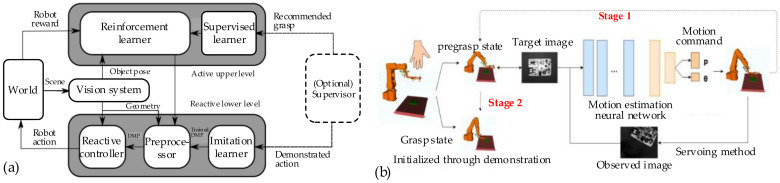
A priori experience-based grasping approaches: (**a**) the hierarchical controller architecture [122], (**b**) the active learning architecture [125].

For unknown objects, there is not enough feature information for robotic grasping. It is difficult to precisely solve the parameters in the process of grasping. As a result, the robotic grasping of unknown objects is hard to carry out by traditional approaches, such as form-closure approaches, force-closure approaches, feedback control approaches, etc. When humans operate on unfamiliar objects, they usually deal with them according to their experience of familiar objects. Similarly, a priori experience is also significant for a robot. To generalize these experiences, many studies focus on using a learning algorithm to train the previous data and adapting these a priori experiences to new unknown objects. In the paper [122], a hierarchical controller based on active learning and reactive control is proposed for global perception and local perception. The controller architecture is shown in Figure 14a. The upper level is used to select the place for object grasping by a reinforcement learner. The lower level consists of an imitation learner and a vision-based reactive controller, which is used to determine the appropriate grasping motions. In order to improve the grasping quality, the grasping function is usually trained by CNN [123]. In the research [124], the Baxter robot is used to establish datasets with more than 50 K data points through 700 h of experiments, and then a CNN is trained to predict the grasp function. By smoothing the grasping function with the pose uncertainty function, the robustness for unknown objects is improved.

In addition, Fu et al. [125] presented an active learning architecture for some accurate industrial manipulation. This approach combines the high accuracy of geometry-driven grasping approaches and the generalization ability of data-driven grasping approaches. As shown in Figure 14b, this process is categorized into the pre-grasping state and the grasping state. In the pre-grasping state, the manipulator actively moves until the object is perceived, and a trained CNN is used to estimate the motion. In the grasping state, the manipulator executes an expected motion to finish the grasping task. Moreover, Quillen et al. [126] proposed a deep reinforcement learning algorithm for grasping policy learning. Off-policy learning enables the utilization of grasping data over a wide variety of objects and improves the ability of generalization to new unknown objects. In order to reduce the cost of training data, an efficient learning algorithm is proposed for robotic grasping [127]. The geometric consistency between the object images and the task space is exploited. A relatively small, fully convolutional neural network is used to predict grasping parameters. The grasping success probability is predicted by a trained learning network for the task space motion [128]. This trained network does not need camera calibration or the current robotic pose. The grasping process is less dependent on the environment and the object—that is, it is more robust with unstructured environments and unknown objects. Although the learning network improves the grasping ability of unknown objects by training with a priori grasping data, the ability of this type of approach to resist external disturbance is insufficient, especially for unknown moving objects and scenarios with high requirements of manipulation accuracy.

**Figure 15:**
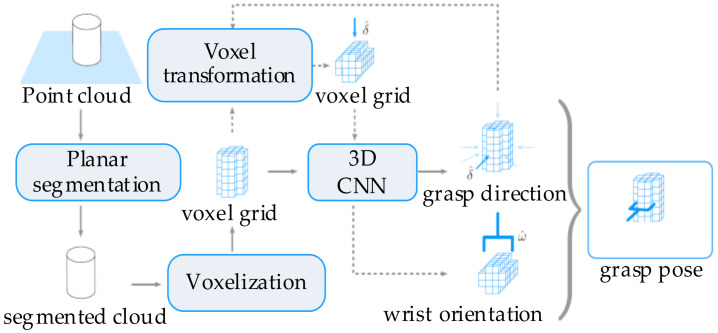
Framework of point cloud processing [133].

**Figure 16:**
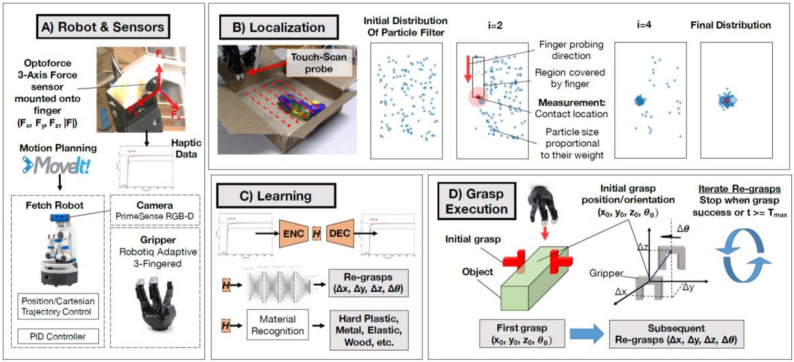
A grasping approach based on sensors and learning networks [134].

Sensors such as cameras, depth sensors, and tactile sensors could be further used to obtain information supplements for the robotic grasping status [129]. Features such as position, shape, pose, and other physical properties provide criteria for the configuration of grasping parameters. For instance, the active vision obtains the object’s contour curvature and updates the robotic pose to acquire the appropriate configuration by maximizing the curvature value [53]. Moreover, the tactile feedback provides tactile feedback for the robotic motion, while vision and other sensors are supplementary [130,131]. For unknown objects with occlusion, the point cloud data could be used to realize the active recognition of objects and then be applied for the semantic segmentation of objects [90,132]. The 3D deep CNN [133], as shown in Figure 15, learns effective features from point clouds and classifies objects by classifier. The grasping direction and wrist orientation are also predicted based on the shape and pose of the object. A typical grasping approach [134] based on sensors and learning networks is shown in Figure 16. In this approach, a touch localization model sequentially explores the workspace and uses a particle filter to aggregate beliefs from multiple hits of unknown objects. The object’s location is estimated, and an initial grasp is generated base on the position of the unknown object. An unsupervised auto-encoding scheme is used to learn the representation of tactile signals. Then, the re-grasping model learns to progressively improve grasps with tactile feedback. This network could estimate the grasp stability and predict the adjustment for the next grasp. Although the tactile-based approach improves the perception accuracy of unknown objects, this is at the cost of high resolution and high time consumption and is not suitable for occasions with high real-time requirements.

## 5. Discussion and Conclusions

In this paper, we review a series of approaches that focus on the feature sensing and robotic grasping of objects with uncertain information. A categorization of uncertain objects based on the type of uncertain information is defined, and uncertain objects are classified into three categories, which are geometric-uncertain objects, physical-uncertain objects, and unknown objects. Based on this classification, we summarize the corresponding approaches for each type of object to present a detailed overview of the feature sensing and robotic grasping of objects with uncertain information. Table 1, Table 2 and Table 3 summarize all the approaches discussed in this paper. Since there are differences between the robot platforms, the properties of the object, and so on in these works, it is meaningless to declare which is better or worse. However, some facts are discussed as follows.

**Geometric-uncertain objects:** For robotic grasping, the object’s geometric information—such as position, shape, pose, etc.—determines how the robot plans its path and grasps the object with a feasible pose. The reviewed approaches of feature sensing and robotic grasping are presented Table 1. The commonly used approach is to collect the object’s images and then detect the position, pose, and other information of the object through the image processing algorithm [17]. The image-based approaches [22,24] achieve the plane localization and space localization of the object, which are used in a variety of situations. The learning networks [32] and CAD models [36,37] obtain the better identification of the shape of the object in large-scale photo collections. For the pose estimation of the object, approaches such as LINEMOD [38], the PCOF-MOD template [40], Hough Forest [41,42,43], Point Pair Features [44,45] and Random Forest [46] can predict the object’s pose quickly and robustly, even if there is partial occlusion. In addition, the point cloud and tactile data are also used to detect the object’s geometric features. The point cloud-based approach could realize the 3D reconstruction of the object, which enables the robot to find the spatial grasping point of the object. The high-resolution tactile sensor [135] could obtain more real feature information of the object, while the detection efficiency is not enough and not suitable for scenarios with a high real-time requirement. In contrast, the image-based approach is more efficient and obtains a better recognition effect. 

In the existing applications, the geometric-uncertain object is usually uncomplicated or commonly encountered. It is not a big challenge to grasp this type of object for a robot. Considering the different requirements, varied structures of graspers are designed. The robot could grasp the object with a feasible grasper structure and predefined parameters according to the specific scenario. The classical approaches, such as form-closure grasp [7] and force-closure grasp [8], can work well for this type of object. As noted previously, varied graspers are designed to adapt the robotic grasping, such as multi-fingered graspers [50,51] and soft graspers [48,54], which enhance the grasping capability for the geometric-uncertain object.

**Physical-uncertain objects:** Compared with geometrical-uncertain objects, the feature sensing and robotic grasping of physical-uncertain objects are more complex. As for the physical properties, such as mass, rigidity, texture, and so on, it is more difficult to build an accurate sensing model due to the applied limitation of specific sensors in robotic grasping. Due to the limitation of effective sensors, the robot is unlikely to directly and accurately detect the object’s mass. Although force/torque sensors [57,59] and tactile sensors [60] have been tried to detect the object’s mass, the detection accuracy depends more on the mathematic model. In addition, approaches based on the geometric outline-mass model [62] and volume-mass model [63,64] also offer new possibilities for mass detection. The rigidity is another challenging property that remained to be detected. Tactile sensors [66,67] could be used to detect this property; however, an unexpected change in the object’s geometric features or damage of the object should be avoided. When it comes to texture detection, approaches are becoming more diverse. A tactile sensor array could be used to detect the object’s texture, while the sensor array has high-resolution requirements. In addition, the texture could also be sensed through image processing. As mentioned in [69,70], the texture of objects in light and dark backgrounds or low-texture and high-light objects can be detected with high resolution. The DMCA [72] learns visual images and tactile data through deep neural networks and achieves cloth texture recognition. The texture detection only with visual perception is easily affected by the image quality, especially the resolution, color difference, and distortion. Multi-sensor fusion can effectively improve the accuracy and robustness of texture detection.

The mentioned physical properties play a major role in the robotic grasping of the object. For instance, the robot needs to grasp the object with an appropriate grasping force so that the object is grasped tightly without slippage and damage. The object’s mass and texture provide the reference for the selection of grasping force. Furthermore, the object’s rigidity determines whether the object is a rigid object or a deformable object. For rigid objects, the deformation of objects is negligible. The LfD approach [76,78] could transfer the manipulation skill from human to robot, which enables a robot to manipulate objects with more flexibility. Skill-based programming [80,81,82,83,84] eases the robot program generation and enables the robot to complete varied object manipulation, such as grasping, picking, and assembly. This approach requires mastering in advance what the robot can accomplish. Moreover, the task-oriented network [86,88] builds a close relationship between object grasping and aimed tasks, which makes tasks easier to be completed by a robot. When it comes to deformable objects, the deformation of objects must be taken into account in the process of robotic grasping. Based on their geometry, deformable objects are classified into three categories, which are linear objects, planar objects, and 3D objects. The robotic grasping and manipulating approach for each type of deformable object are listed in Table 2. Most of the approaches are only applicable to specific objects; the generalization ability is not enough. In addition, the nonlinear physical model of deformation needs to be studied and the real-time performance also needs to be improved to adapt the time requirements of the working situation.

**Unknown objects:** Since there are too many uncertainties about the geometric and physical features of the object, this type of object is the most challenging for a robot to handle. In order to sense the object’s feature, the first step is to search and localize the object. As showed in Table 3, the reviewed approaches are classified into three categories, which are image-based approaches [107,108], point cloud-based approaches [109], and tactile perception-based approaches [110,111,112]. The first two approaches are relatively mature and could deal with the problem of multi-object search in a cluttered environment. The POMDP [108], extremum seeking strategy [107], and learning networks [109] are usually used in these approaches and the results are acceptable to a certain extent. However, these methods require a higher quality of images and point clouds. When the important features of the object, such as texture and shape, are obscured, the position accuracy may greatly decrease. In contrast, the tactile perception-based approach obtains the realistic position information of unknown objects through the sense of touch, as humans do. The decision-theoretic approach [110] guides robotic actions by tactile feedback to search the object, while the Bayesian-based approach [111] can solve the 6DoFs localization problem by the measurement of contact points. Moreover, the active approach proposed in [112] enables the robot to explore the whole workspace and calculates the 3D minimum bounding box of the object. However, the efficiency of this approach is not enough, and it is difficult to establish an accurate analytical model to evaluate the relationship between tactile data and object features.

As the object’s position is obtained, the robot needs to identify more features of the object to support the robotic grasping. The approaches used for geometric-uncertain objects and physical-uncertain objects could also be extended to unknown objects after the identification model is redesigned and optimized. For example, the MOPED [115] based on the Iterative Clustering Estimation algorithm can complete the multi-object pose estimation and detection. It addresses two main challenges, which are robust performance in complex scenarios and low latency for real-time operation. In addition, any characteristic of unknown objects is also valuable for object recognition. For instance, the unknown object’s color data from color sensors are helpful for the recognition of the object’s shape and pose [119,121]. The material is also significant for the identification of unknown objects; however, there is no highly effective way to detect the category of material—such as metal, plastic, glass, etc.—in the robotic field. There are two possible assumptions, which are Non-Destructive Testing Technology (NDTT) [136,137] and Ray Transmission Imaging Technology (RTIT) [138,139,140] and may be used to classify the material. In general, the NDTT is used for product defect detection; however, it may also be used for material detection. For instance, ultrasonic testing is an NDTT and is able to detect metal, nonmetal, and composite materials. The classification model needs to be further studied, and it identifies the type of material based on ultrasonic signals. The RTIT is usually used in security check occasions, especially in airports, stations, etc. The object’s shape and density information are easily obtained through the RTIT. Before identification, a material matching database needs to be established firstly, and then the category of material is classified based on the a priori knowledge of material categories.

Although some features of unknown objects could be acquired through feature sensing, there are still errors between these features and real features. As a result, the robotic grasping approaches for familiar objects are not applied to unknown objects, and even approaches for geometric-uncertain objects and physical-uncertain objects are also hardly used in the robotic grasping of unknown objects. Inspired by humans, the grasping experience of familiar objects is significant for the grasping process of unknown objects. These experiences could improve the robustness of the grasping ability to uncertain properties. Learning-based approaches, such as active learning [125], deep reinforcement learning [126], and 3D deep CNN [133], play a significant role in experience transferring and are becoming a future trend to be more investigated. However, the learning-based approach is usually time-consuming because of the training and learning process based on a large database. As a result, it not suitable to deal with the sudden change in objects at industrial lines. Additionally, it does not overcome the object’s uncertain features. When it comes to the high-precision scenario, such as peg-in-hole assembly with small clearance, these uncertain features may still have a converse impact on the accuracy of subsequent manipulation tasks. To solve this problem, a feasible way is that multiple types of sensors are used to provide more abundant information about the object. Especially with the development of sensor technology, the grasping approach based on learning networks and multi-sensor fusion has attracted more attention. A typical approach is the sensors and learning networks-based approach [134]. This type of approach is that the learning network generates an initial grasping configuration and the additional sensor is used to provide feedback information to correct the process of grasping. For instance, as the object is grasped based on the learning work, the grasper with finger tactile sensors detects the grasping state in real time. Then, the robot timely adjusts the grasping configuration according to the grasping state, which is helpful to improve the accuracy of the manipulation task. This way requires high real-time performance, which, in turn, imposes more requirements of the robot’s hardware and the complexity of the algorithms.

Hopefully, this review will help researchers with the feature sensing and robotic grasping of objects with uncertain information. Since there are many differences between the test platform and the feature type of uncertain objects, it is impossible to choose an approach that has a good performance for all uncertain objects. For the grasp and manipulation of uncertain objects, there are several interesting problems to be more investigated in the future, including (a) the effective sensing approach for object’s unconventional features, such as irregular shape and real-time deformation; (b) the precise correlation model between object grasping and task manipulating; and (c) the real-time evaluation criteria for the degree of object grasping and task completion.

## Figures and Tables

**Table 1:** Feature sensing and robotic grasping for geometric-uncertain objects.

**Sensing**	Position detection	2D images [17,22], 3D images [23,24], Point clouds [18,25,26], Spatial-temporal feature [27,28,29,30], Sensory-fusion feature [31]
	Shape identification	Learning-based [32], 2D boundary [33], 3D class model [34], FCNs and CRFs [35], Dense CAD model [36], DeformNet [37]
	Pose estimation	Template-based approaches (LINEMOD [38], Hierarchical fragment matching [39], PCOF-MOD template [40]); Voting-based approaches (Hough Forest [41,42,43], Point Pair Features [44,45]); Learning-based approaches (Random Forest [46], Deep quadruplet CNN [47])
**Grasping**	Direct configuration-based grasping	MDISF & GTO [52], Active vision [53], Underactuated tendon-driven [54]

**Table 2:** Feature sensing and robotic grasping for physical-uncertain objects.

**Sensing**	Mass estimation	Force and torque [57], 3D force vector [59], Deep learning and STAG [60], Geometric outline-mass model [62], Volume-mass model [63,64]
	Rigidity prediction	Motion analysis model [65], Flexible tactile-sensor array [66,67]
	Texture detection	Higher order statistics [68], Edge-texture feature [69], Texture rendering model [70], Texture contour model [71], DMCA [72]
**Grasping**	Rigid objects	LfD(CC-LfD [76], RGB-D observation-based demonstration [78]); Skill-based programming [80,81,82,83,84]; Task oriented-based grasping (RGB-D part-based [85], BADGr [86], TOG-Net [88])
	Deformable objects	Linear objects (Robotic individual skills [91], Flexible rope model [92]); Planar objects (Predefined parameter [95], Fiducial marker tracking [96], Mass–spring–damper model [97], Extended deformable model [98,99]); 3D objects (Point clouds [101], Tactile data [102,103], Kelvin–Voigt model [104], Finite-element model [105])

**Table 3:** Feature sensing and robotic grasping for unknown objects.

**Sensing**	Search & localization	Image-based approaches (Extremum Seeking Control [107], POMDP [108]); Point cloud-based approaches (PointFusion architecture [109]); Tactile perception-based approaches (Decision-theoretic approach [110], Bayesian-based approach [111], Active exploration approach [112])
	Pose estimation	DenseFusion [113], CNN [114], MOPED [115]
	Shape detection	Information Gain Estimation [116], Monte Carlo tree [118]
	Other properties identification	Color (Color descriptors [120], Hi-Erarchical Temporal Memory [121]); Mass (Approaches used for physical-uncertain objects could be extended to unknown objects); Material and Compactness (Not a very effective approach)
**Grasping**	Learning-based grasping	Hierarchical controller [122], Active learning [125], Deep reinforcement learning [126], 3D deep CNN [133], Sensors and learning networks-based approach [134]
